# Evaluation of humoral immune responses to enterotropic lentogenic VG/GA vaccine of Newcastle disease in commercial turkey poults (*Meleagris gallopavo*)

**DOI:** 10.1186/s13028-019-0476-y

**Published:** 2019-08-27

**Authors:** Gholamreza Nikbakht Brujeni, Mohammad Hassanzadeh, Hassan Al-Karagoly, Tohid Tolouei, Atefeh Esmailnejad

**Affiliations:** 1 Department of Microbiology and Immunology, Faculty of Veterinary Medicine, University of Tehran, Tehran, Iran; 2 Department of Avian Diseases, Faculty of Veterinary Medicine, University of Tehran, Tehran, Iran; 3 Department of Internal and Preventive Medicine, Faculty of Veterinary Medicine, University of Al-Qadisiyah, Al-Qadisiyah, Iraq; 4 Department of Pathobiology, School of Veterinary Medicine, Shiraz University, Shiraz, Iran

**Keywords:** Newcastle disease, IgA, IgY, Turkey, VG/GA vaccine

## Abstract

**Background:**

*Villegas*-*Glisson*/University of Georgia (VG/GA) strain of Newcastle disease virus (NDV) is recommended for the initial vaccination of commercially reared turkey poults. However, the vaccine-induced antibody responses have not been studied in this species. The level of systemic humoral immune responses against the NDV was investigated in commercial turkey poults vaccinated with the VG/GA vaccine. One hundred eighty-two hybrid strain of turkey poults (*Meleagris gallopavo*) were divided randomly into vaccinated and unvaccinated groups. The vaccinated group was given the VG/GA vaccine at 10 and 20 days of age. To investigate the vaccine immunity, the level of specific IgY and IgA in serum samples were determined using ELISA and haemagglutination inhibition assays (HI). The biological half-life of maternal antibodies was also determined before the immunization.

**Results:**

VG/GA-specific antibodies were detected in the vaccinated turkey poults and were significantly higher in the vaccinated group compared to the unvaccinated group. IgY and IgA antibodies showed a significant increase in titers 14 days after the second vaccination and reached a peak on day 35 of age. The correlation coefficient and intra-rater reliability showed a significant correlation between the HI titers and IgY/IgA ELISA values. Maternal IgY and IgA levels were found to decline in the serum with half-lifes of 7.68 ± 2.35 and 2.18 ± 0.82 days, respectively.

**Conclusions:**

Enterotropic lentogenic VG/GA vaccine induced a marked humoral immune response against the NDV in turkey poults. The positive correlation between IgY and IgA highlights the role of these two antibody classes in controlling the Newcastle disease in turkey poults.

## Background

Newcastle disease (ND) is a highly contagious viral disease that represents a considerable threat to the poultry industry all over the world. It is caused by Newcastle disease virus (NDV) which has been designated as type 1 of avian avulavirus [1]. Avian species differ in terms of their susceptibility to the ND, and the virus strain also plays an important role in the severity of the disease. Based on the severity of disease induced, NDV strains are classified into lentogenic, mesogenic, and velogenic strains [2–4]. Dyspnea, gasping, cyanosis of the comb, wattle, crop dilatation, catarrh, foamy mucus in the pharynx, ataxia, paralysis, and torticollis are the most obvious signs of ND that affect the respiratory tract, circulatory system, gastrointestinal tract, and nervous system [5].

A direct correlation between antibody response and the transmission potential of the disease in susceptible birds highlights the role of specific antibodies in controlling ND [6]. Therefore, the monitoring of successful vaccination in flocks demonstrated by an elevation in the antibody titer within a few days after the vaccination is pivotal. In addition, the measurement of the level of specific antibodies has proven to be of great value in assessing herd immunity and critical vaccination coverage [7, 8]. Beside the routine titration of serum IgY antibodies, it is important to evaluate the systemic IgA response since that can help us predict the secretory IgA level and verify the correlation between the serum antibody level and the specific IgA response in local secretions [9, 10].

Immune responses and the efficacy of vaccination by *Villegas*-*Glisson*/University of Georgia (VG/GA) strain of NDV has been evaluated in commercial broiler chickens [11–13]. Since VG/GA vaccine elicits an adequate immunity with minimal respiratory reactions in chickens, it is also used in turkeys. However, the VG/GA vaccine-induced antibody responses have not been studied in this species. In the present study, the level of systemic humoral immune responses (IgY and IgA) against the NDV was investigated in commercial turkey poults vaccinated by the VG/GA strain of NDV vaccine.

## Materials and methods

### Study design

One hundred and eighty-two 1-day-old male commercial turkey poults (*Meleagris gallopavo*), hybrid strain (French origin), were obtained and kept for 42 days in the experimental pens. The poults were kept in concrete floor pens covered with 8 cm of clean pine wood shavings, and each pen was equipped with one tube feeder and one automatic drinker. Throughout the study, the poults were kept following standard temperature regimens, which gradually decreased from 38 to 23 °C and under 16L:8D light schedule. Birds were allowed to consume feed and water ad libitum. All birds were marked with individual numbers on tags. Blood samples were collected from all the poults at 1 and 9 days of age for quantitation of maternal antibody levels. The breeding conditions were approved by the Tehran University Policy on Animal Care and Use.

### Vaccination

At 10 days of age, the turkey poults were divided into vaccinated (n = 92) and unvaccinated (n = 90) groups. The vaccinated group was given the VG/GA vaccine at 10 and 20 days of age by eyedropper. One dose of vaccine (1 mL), containing 106.5 EID^50^ (50% Embryo Infectious Dose) of the lentogenic freeze-derived live VG/GA strain of NDV (Merial Animal Health Limited, Lyon, France), was used for each animal. The unvaccinated group was treated similarly with sterile PBS. Blood samples were collected from the experimental groups at days 20, 28, 35, and 42 of age, centrifuged to separate the sera, and stored at − 20 °C until further analyses.

### Enzyme-linked immunosorbent assay (ELISA)

The optimization of the VG/GA-specific antibody titer and specificity was conducted using a modified enzyme-linked immunosorbent assay (ELISA) [14]. The VG/GA vaccine was used as coating antigen and the checkerboard titration procedure used for determination of the optimal concentration/dilution of antigen and antibody. Polystyrene microtiter plates were coated with 50 μL of diluted antigen (VG/GA), ranging from 1.5 to 100 µg/mL (serial fourfold dilution) and incubated at 4 °C overnight. Unbound antigens were removed by washing them with PBS-T (PBS containing 0.05% [vol/vol] Tween-20), and uncoated surfaces were blocked using 200 μL/well of PBS-Blotto (PBS containing 0.5% [wt/vol] nonfat dry milk) at 37 °C for 1 h. After three additional washing steps, 100 μL of pooled serum, collected from immunized group (30 birds), was added to the wells using twofold dilutions starting at 1:20 and left for 30 min at 37 °C. Pooled samples were collected from vaccinated group at day 35, 2 weeks after second vaccination. The plates were then washed three times and incubated with 100 μL/well of diluted (1:10,000) horseradish peroxidase (HRP)-labeled goat anti-chicken IgY/IgA (Biorad, Oxford, UK) for 30 min at 37 °C. The plates were washed an additional three times and a color reaction was initiated by adding 100 μL/well of tetramethyl benzidine (TMB) (Sigma Aldrich, St. Louis, MO, USA) substrate and incubated at room temperature for 15 min. The reaction was stopped by adding 100 mL/well of stop solution (3 M H_2_SO_4_), and the optical density (OD) of each well was determined at 450 nm with a microtiter plate reader (Stat FAX 2000, Awareness Technology, Inc., USA).

According to the data obtained from the checkerboard titration, coating antigen optimal concentration was 7 µg/mL for IgY and 30 µg/ml for IgA test plates. Serum samples were tested at 1:100 dilution for IgY and 1:20 for IgA antibodies. All the above-mentioned protocol for checkerboard titration was followed for both antibodies. The same prepared pooled serum sample from immunized group (30 birds) was titrated in 12 serial dilutions (twofold). Antibody end-point titer of a pooled serum sample was expressed as the reciprocal of the highest dilution that gave a reading above the blank absorbance value. By using a pooled serum sample in each run, a 7-point (twofold dilution) standard curve was performed and some equations were derived from the linear regression analyses. The end-point titer of each serum sample was then calculated using its absorbance value in the standard curve equation, obtained from a pooled serum sample which was incorporated in each plate.

### Haemagglutination inhibition assay (HI)

The antibody response against the VG/GA vaccine was also determined by performing a standard haemagglutination inhibition assay (HI) using the VG/GA virus as antigen. Briefly, PBS (25 µL) was dispensed into 12 wells of one row of v-bottomed microtiter plates. Four haemagglutinating units of the virus were added into each well and the mixture was incubated at room temperature for 30 min. Then, 1% turkey erythrocytes were added to each well and allowed to settle down for 40 min at room temperature. To extract nonspecific inhibitors, serum samples were pretreated at 56 °C for 30 min, incubated with kaolin 5% (weight/volume) at 37 °C for 30 min, and then added to the plate. A duplicate twofold dilution series of each sample was prepared and HI titers were calculated as log_2_ value to express the titers of the highest reciprocal of the dilution.

### Statistical analysis

All data were expressed as mean and standard errors of the mean (SEM). Data were analyzed statistically using Student’s t-tests for pairwise comparisons of IgY/IgA responses between the vaccinated and control groups. Repeated-measures ANOVA was used to test any significant differences in the antibody responses measured at different specific time points. Variability in the immune responses to the VG/GA vaccine was also measured among the turkey pullets as the analytical coefficient of variation (CV%). Fisher’s *r*-to-z conversion of correlation coefficients was used to obtain the *P* values in correlation analysis among the immune responses. Kappa (κ) was calculated to measure the strength of the correlation between the HI titers and ELISA values. The biological half-life of maternal Newcastle-specific antibodies was also calculated as described by King et al. [15]. A probability of P ≤ 0.05 was considered statistically significant. Data were analyzed with SPSS software, version 16 for Windows (SPSS Institute, Chicago, IL, USA).

## Results

### Maternal antibody titer

The maternal antibody titers were tested in samples from turkey poults at 1 and 9 days of age, before vaccination. The HI titers and IgY/IgA mean titer values decreased at 9 days of age compared to day 1. The half-life of the maternal antibodies in the serum samples was also determined. Maternal IgY and IgA levels were found to be eliminated in the serum with a half-life of 7.68 ± 2.35 and 2.18 ± 0.82 days, respectively (Table 1).

**Table 1:** VG/GA specific antibody levels measured at 1 and 9 days of age in turkey poults

	HI			IgY			IgA		
	Mean	SD	CV%^a^	Mean	SD	CV%^a^	Mean	SD	CV%^a^
Day 1	7	1.41	20.14	654.95	94.72	14	74.84	16.96	23
Day 9	4.85	1.18	24.32	330.67	78.45	24	25.73	9.63	37
Half-life (day)	3.19	1.4	43.89	7.68	2.35	31	2.18	0.82	38

^a^Coefficient of variation

### Antibody responses to vaccination

The HI titers, IgY, and IgA levels at different specific time points after vaccinations are summarized in Table 2. Specific antibodies were detected in all the immunized poults. Throughout the experiment, these antibodies were significantly higher in the poults in the vaccinated group compared to the unvaccinated group (P < 0.001).

**Table 2:** Descriptive statistics of the HI titers and the IgY/IgA ELISA values against the VG/GA vaccine in turkey poults

	Days	Unvaccinated group			Vaccinated group		
		Mean	SD	CV%^a^	Mean	SD	CV%^a^
HI	20	3.89	1.33	34.19	1.6	0.8	50
	28	2.98	0.11	3.69	1.17	0.38	32.48
	35	6	1.42	23.66	1	0.18	18
	42	6.2	1.39	22.42	1.03	0.18	17.48
IgY	20	1011.12	179.6	17.76	464.7	122	26.25
	28	1366.88	221.06	16.17	488.51	78.67	16.1
	35	1586.45	224.38	14.14	459.06	114.94	25.04
	42	1157.61	156.34	13.51	303.8	63.5	20.9
IgA	20	179.31	56.4	31.45	104.65	12.32	11.77
	28	381.69	34.43	9.02	59.6	8.91	14.95
	35	398.7	138.64	34.77	60.7	12.48	20.56
	42	374.51	87.38	23.33	59.53	15.19	25.52

^a^Coefficient of variation

**Fig. 1:**
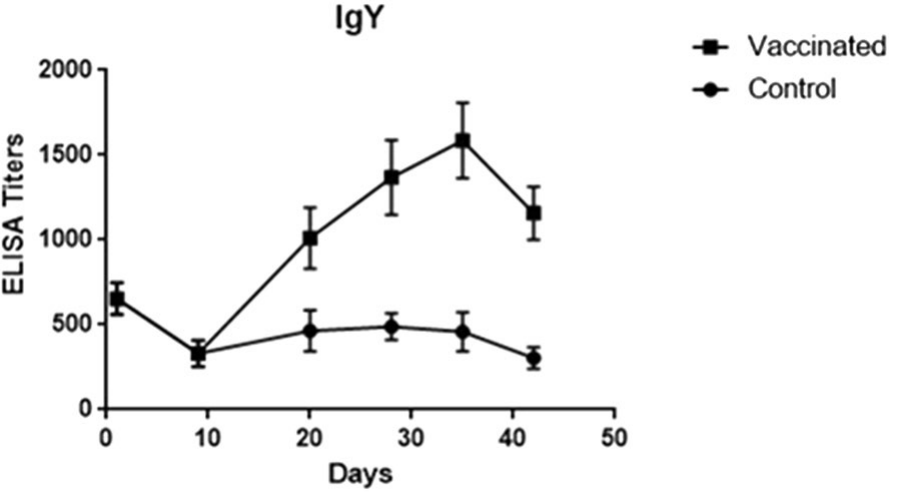
IgY titers at different specific time points after vaccinations. Specific antibody against the VG/GA antigene was measured using ELISA in all the immunized poults and control group. Samples collected from the experimental groups at days 0, 10, 20, 28, 35, and 42 of age

**Fig. 2:**
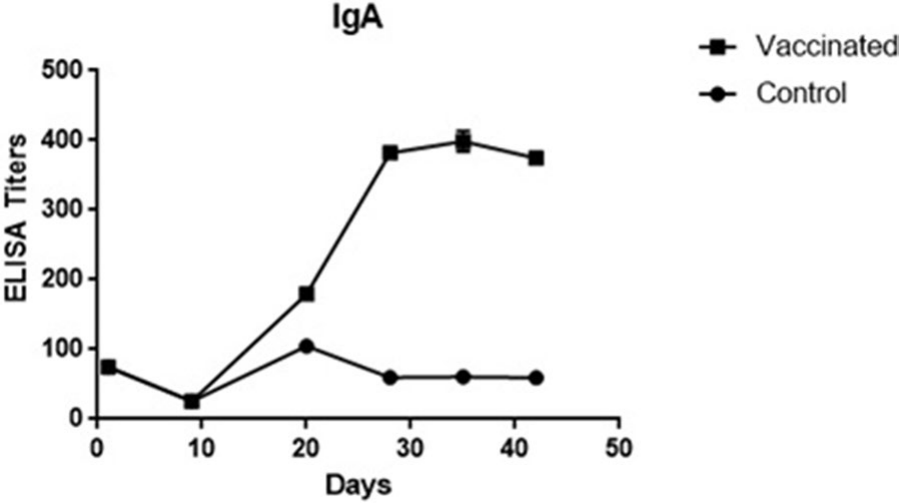
IgA titers at different specific time points after vaccinations. Specific antibody against the VG/GA antigene was measured using ELISA in all the immunized poults and control group. Samples collected from the experimental groups at days 0, 10, 20, 28, 35, and 42 of age

VG/GA-specific IgY and IgA were detected towards day 20 of age, 10 days following the first immunization. Both IgY and IgA antibodies showed a significant increase in titer 14 days after the second vaccination and reached a peak at day 35 of age. IgY showed a significant increase only after the first vaccination, but the IgA titer increased dramatically after the first and second vaccinations. However, a slight decrease was observed in the levels of both antibodies at day 42, with the decrease not being statistically significant (Figs. 1, 2).

**Fig. 3:**
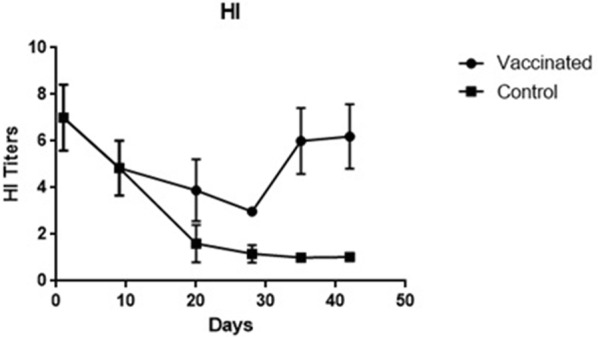
HI titers at different specific time points after vaccinations. The antibody response against the VG/GA vaccine was determined by performing a standard haemagglutination inhibition assay (HI) using the VG/GA virus as antigen. HI titers were calculated as log2 value to express the titers of the highest reciprocal of the dilution

Based on the HI tests, antibody titers continued to decrease up to 28 days of age and was significantly increased by day 35. The HI titer increased from 3.891 ± 1.330 after the first vaccination (day 20) to 6.196 ± 1.385 at 3 weeks after the second vaccination (day 42) (Fig. 3).

The variability in the immune responses to the VG/GA vaccine (HI titer and IgY/IgA values) was also measured among the turkey poults and expressed as the analytical coefficient of variation (CV%). There was a considerable variation (CV ≥ 20%) in HI titer, IgY, and IgA levels before vaccination (Table 1). The individual variations decreased significantly in the IgY level after the second vaccination (Table 2).

### Correlation between HI, IgY, and IgA titers

**Fig. 4:**
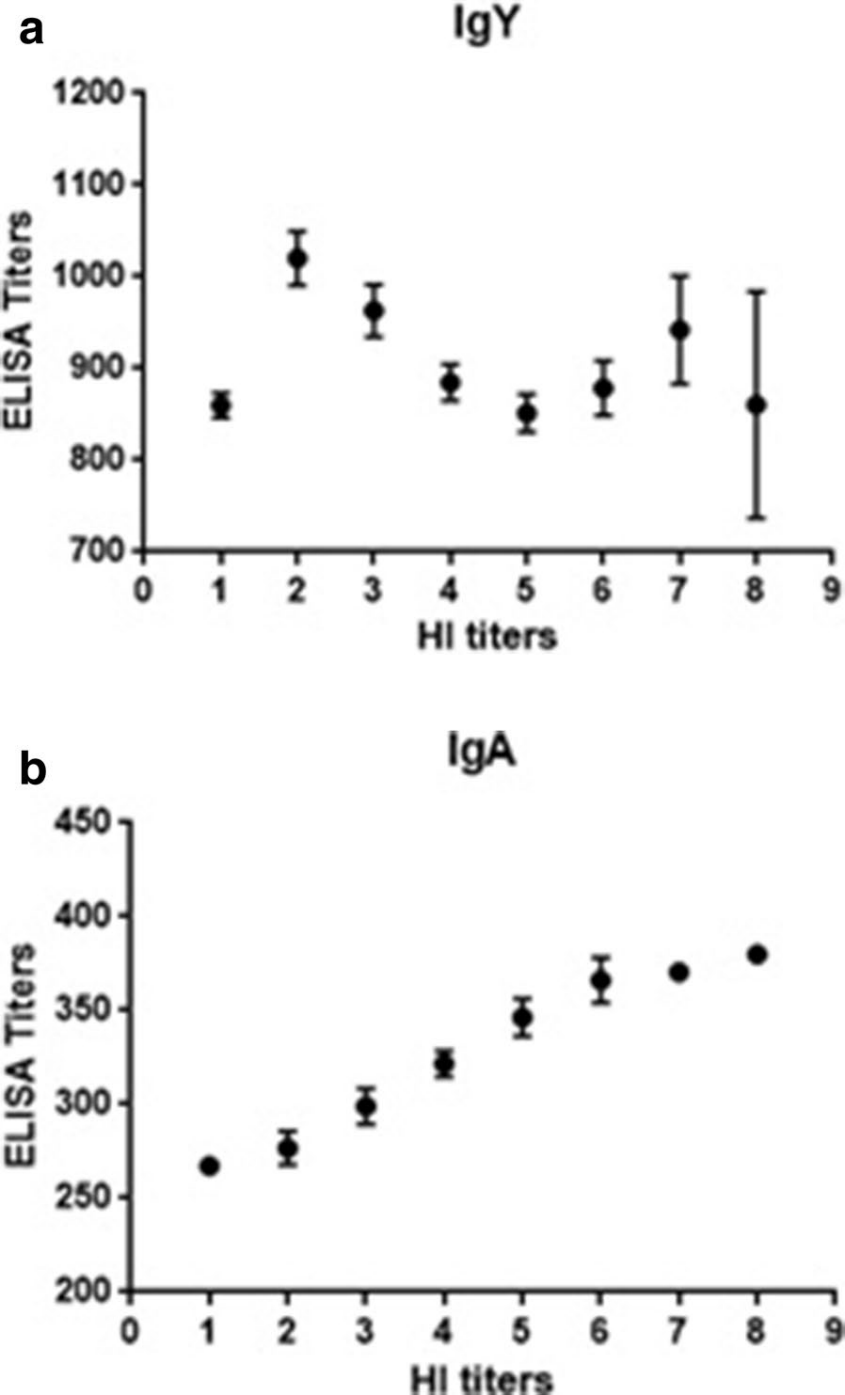
Correlation between HI titers and IgY/IgA ELISA values. **a** Correlation between IgY values obtained by ELISA in each HI titer group. **b** Correlation between IgA values obtained by ELISA in each HI titer group

The correlations between the HI titers and IgY/IgA ELISA values were evaluated in this study (Fig. 4a, b). In total, a significant association was observed between HI titer and IgA level in turkey poults (P < 0.05). However, correlation between HI titer and IgY value obtained by ELISA, was only significant in the range of 0 to 2 of HI titer (log_2_ ≤ 2).

Analyzing the correlation between the antibody titers measured at different time points showed a significant positive correlation between IgY and IgA titers at 28 days of age (*r *= 0.213, P ≤ 0.042). No significant correlation was detected between the HI titers and IgY levels after the first and second vaccinations up to day 28. After that, a significant association was observed between HI and IgY titers at 35 (*r *= 0.912, P ≤ 0.012) and 42 days of age (*r *= 0.718, P ≤ 0.038). Significant correlations were seen between the HI titers and IgA levels at days 20 (*r *= 0.494, P ≤ 0.0001), 28 (*r *= 0.648, P ≤ 0.0001) and 42 (*r *= 0.16, P ≤ 0.013). The correlation coefficient and intra-rater reliability showed a close correlation between HI titers with IgY (*k *= 0.353 ± 0.0381, P < 0.0001), and IgA (*k *= 0.25 ± 0.022, P < 0.0001) ELISA values.

## Discussion

Different avian species show different patterns of resistance or susceptibility to ND. Variations in the level of immune responses among different populations can be ascribed to several factors, including genetic background, environmental factors, and virus strains [1, 3]. Effective vaccination against ND is determined by the measurement of specific antibodies. The Villegas-Glisson/University of Georgia (VG/GA) strain of NDV is a common phenotype of live mucosal vaccines that follows a pattern similar to that of the natural infection and promotes specific serum IgY and IgA responses in chickens [13, 16]. Although the VG/GA vaccine is also recommended for the initial vaccination of commercial turkey poults, the vaccine-induced antibody responses have not yet been studied in this species.

In this study, the level of systemic humoral immune responses (IgY and IgA) against VG/GA Newcastle vaccine was investigated in commercial turkey poults. The results revealed that the biological half-life of maternal NDV-specific IgY (7.68 ± 2.35) and IgA (2.18 ± 0.82) antibodies were higher in turkey poults in comparison to that of the commercial broiler chickens [17]. While the IgY level increased ∼ threefold following the first vaccination, no significant increase was observed in the IgY titer after the second vaccination. The level of IgA increased ∼ sixfold immediately following the first vaccination and this continued after the second immunization (∼ twofold). In contrast to the broiler chickens, no decreases were observed in the antibody levels after immunizations in turkey poults [18–20]. Although the HI titer steadily decreased by the day 28 of age, it was elevated significantly following the second immunization. The data obtained from the turkey poults were in close agreement with those of the previous studies which revealed an increase in the antibody titer at 14 to 21 days post vaccination in chickens [21]. It has been demonstrated that NDV-specific antibodies can be detected in chickens’ blood, starting at the first week after the infection or live virus vaccination and reaching a peak 21 to 28 days post immunization [8]. An increase in the IgY titer was also observed at 7 days after the primary vaccination and 7 to 14 days after the booster vaccination in chickens [21]. In comparison with chickens, the increase and decrease in the VG/GA-specific IgY titer were faster in the vaccinated turkey poults. Interestingly, the IgA titers increased markedly and peaked at day 28 and no significant decrease was detected until day 42.

The study revealed a significant positive correlation between IgY and IgA antibody titers developed in response to the VG/GA vaccine in turkeys. As a result, NDV-specific serum IgY could be considered as a promising indirect marker for the specific IgA response. Comparing the ELISA and HI titers measured at different specific time points showed a significant association between the HI titers and IgY levels at days 35 and 42 of age, following the second vaccination. In the case of IgA, a significant correlation was observed at days 20, 28, and 42 of age. The linear association between the HI titers and IgA ELISA values may suggest that a considerable part of IgA antibodies has HI activity. Indication of no significant correlation between IgY level and HI titers also suggests that a large proportion of the IgY antibodies lack HI activity, but overall both IgY and IgA antibodies have HI activities. Moreover, ELISA titers may reflect the competition between IgY and IgA antibodies for the same viral epitopes. It is worth noting that because of the fair agreement between the HI titers and IgY/IgA ELISA values, neither IgY nor IgA levels could be used alone for a valid prediction of the HI titer.

## Conclusions

Enterotropic lentogenic VG/GA vaccine can induce a marked IgY and IgA immune response against the NDV in turkey poults. The gradual and steady increase in the immune responses indicates that the VG/GA vaccine can induce good immunity in turkey poults.

## Data Availability

The datasets used and/or analyzed during the current study are available from the corresponding author on reasonable request.
